# Supplementary material for: Sodium permeable and “hypersensitive” TREK‐1 channels cause ventricular tachycardia
Source: EMBO Mol Med. 2017 Feb 27;9(4):403–14. doi: 10.15252/emmm.201606690 (PMC5376757; doi:10.15252/emmm.201606690)
Supplement: Supplementary file 1 — Appendix [file EMMM-9-403-s001.pdf]

Appendix

Sodium permeable and `hypersensitive´ TREK-1 channels cause ventricular tachycardia

Niels Decher[1,*,§], Beatriz Ortiz-Bonnin[1,6,§], Corinna Friedrich[2], Marcus Schewe[3], Aytug K. Kiper[1], Susanne Rinné[1], Gunnar Seemann[7,8], Rémi Peyronnet[7,8], Sven Zumhagen[2], Daniel Bustos[4], Jens Kockskämper[6], Peter Kohl[7,8], Steffen Just[5], Wendy González[4], Thomas Baukrowitz[3], Birgit Stallmeyer[2], & Eric Schulze-Bahr[2]

1 Institute of Physiology and Pathophysiology, Vegetative Physiology, Philipps-University of Marburg, Marburg, Marburg, 35037, Germany
2 Institute for Genetics of Heart Diseases (IfGH), Department of Cardiovascular Medicine, University Hospital Münster, Münster, 48149, Germany
3 Institute of Physiology, Christian-Albrechts-University of Kiel, Kiel, 24118, Germany
4 Center for Bioinformatics and Molecular Simulation, University of Talca, Talca, 3460000, Chile
5 Molecular Cardiology, University Hospital Ulm, Ulm, 89081, Germany
6 Institute of Pharmacology and Clinical Pharmacy, Biochemical and Pharmacological Center (BPC) Marburg, Marburg, 35032, Germany
7 Institute for Experimental Cardiovascular Medicine, University Heart Center Freiburg - Bad Krozingen, Medical Center – University of Freiburg, Freiburg, 79110, Germany
8 Faculty of Medicine, University of Freiburg, Freiburg, 79110 Germany

Supplementary contents:

Appendix Fig S1	Page 2
Appendix Fig S2	Page 3
Appendix Fig S3	Page 4
Appendix Fig S4	Page 5
Appendix Fig S5	Page 6
Appendix Fig S6	Page 7
Appendix Fig S7	Page 8
Appendix Fig S8	Page 9
Appendix Fig S9	Page 10
Appendix Fig S10	Page 11
Appendix Fig S11	Page 12
Appendix Fig S12	Page 13
Appendix Table S1	Page 14
Appendix Table S2	Page 15
Appendix Table S3	Page 16
Appendix Table S4	Page 17
Appendix Supplementary Methods	Page 18

Appendix Fig S1

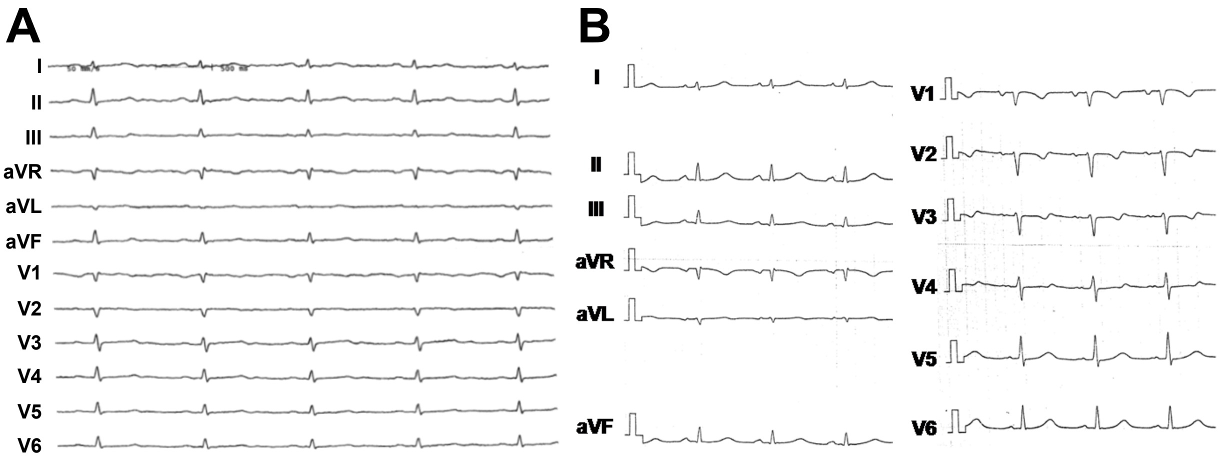

Appendix Fig S1. Resting 12-Lead ECGs of proband 10772-3 with documented right ventricular outflow tract tachycardia (RVOT-VT; Fig 1A).
(A) Normal baseline ECG at 49 years during an invasive electropyhsiologic study. (B) Normal baseline ECG directly after terminated RVOT-VT.

Appendix Fig S2

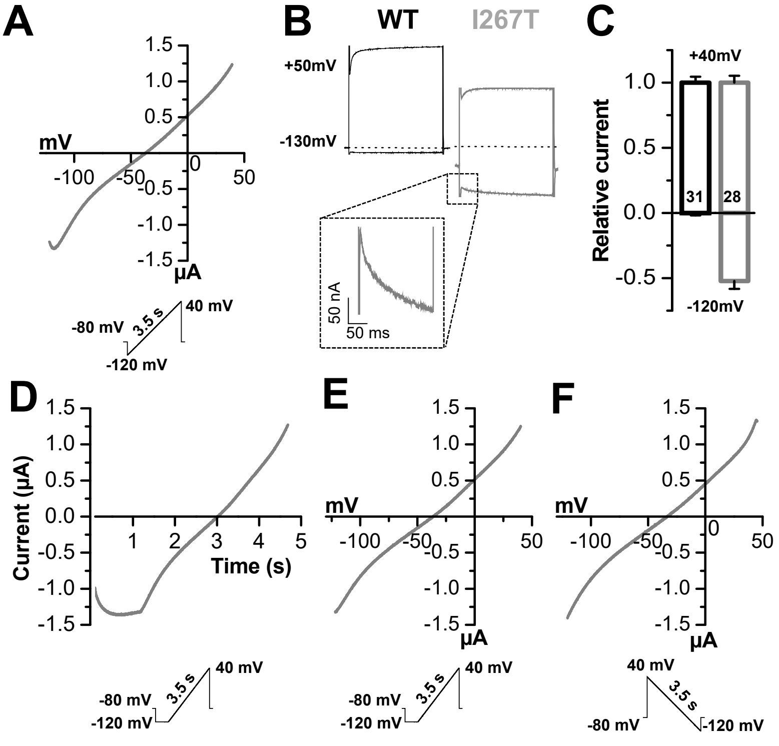

Appendix Fig S2. Current kinetics of the TREK-1^{I267T} mutant.
(A) Representative current-voltage relationship of TREK-1^{I267T}. Voltage was ramped from -120 mV to +40 mV within 3.5 s, the holding potential was -80 mV. (B) Example traces of TREK-1 (*black*) and TREK-1^{I267T} (*gray*) stepping for 200 ms to -130 mV or + 50 mV. The inset shows a magnification highlighting the slow activation kinetics of the I167T mutant, when stepping to hyperpolarized membrane potentials. (C) Mean relative current amplitude of wild-type (*black*) and TREK-1^{I267T} (*gray*) at -120 mV, normalized to the current at +40 mV. Relative current at -120 mV was -0.02 ± 0.00 (n = 31) for wild-type and -0.52 ± 0.06 (n = 28) for TREK-1^{I267T}. (D) Representative current measurement of the TREK-1^{I267T} mutant, recorded from a holding potential of -80 mV, but with a pre-step of 1 s to -120 mV to fully activate TREK-1^{I267T} before the voltage was ramped within 3.5 s from -120 to +40 mV. Note that here time is plotted against the current, to illustrate the activation of TREK-1^{I267T} upon hyperpolarization. (E) The same recording as in (D), plotting the voltage against the current. Here no initial hook can be observed, as there was sufficient time for the mutant to fully activate, before the voltage was ramped. Compare to panel (A) and Figure 2D, were the initial \`hook´ is present, when there is no negative pre-pulse protocol used. (F) Representative current-voltage relationship measurement of the TREK-1^{I267T} mutant, when the voltage was ramped from +40 mV to -120 mV within 3.5 s. Given that the mutant can be activated at depolarized potentials there is no \`hook´ present when the potential is slowly ramped to very hyperpolarized potentials. Data are presented as mean ± SEM.

Appendix Fig S3

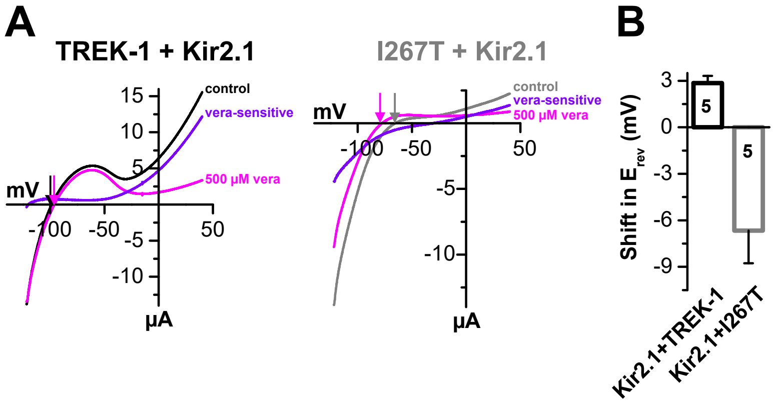

Appendix Fig S3. TREK-1^{I267T} depolarizes the membrane potential in the presence of Kir2.1 inward rectifying potassium channels.

(A) Example traces of Kir2.1 co-expressed with TREK-1 or TREK-1^{I267T}. Block by 500 µM verapamil (vera) is indicated in magenta. The depolarization of the E_{rev} for TREK-1 expressing oocytes (left panel) and the hyperpolarization of the E_{rev} for TREK-1^{I267T} (right panel) are indicated with magenta arrows. The respective verapamil-sensitive currents are indicated in purple. (B) Shift of the E_{rev} in cells co-expressing Kir2.1 and TREK-1 (*black*) or TREK-1^{I267T} (*gray*) after application of 500 µM of verapamil (n = 5). Data are presented as mean ± SEM.

Appendix Fig S4

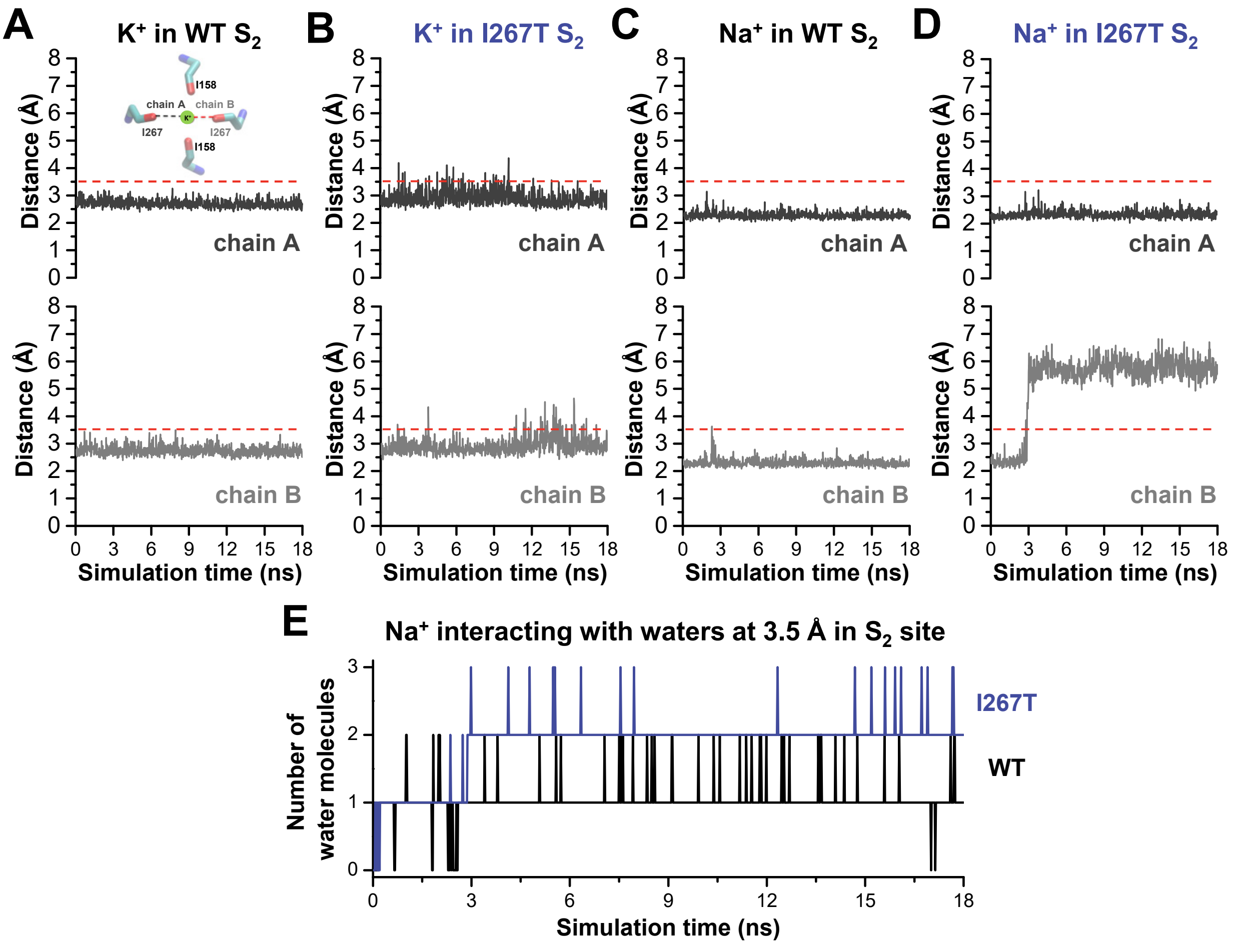

Appendix Fig S4. The oxygen of the I267 carbonyl backbone coordinates K^+ ions in wild-type but less efficient in TREK-1^{I267T}. For I267T more water molecules, instead of oxygen atoms, coordinate the Na^+ ion.

Oxygens within a confinement radius of 3.5 Å around the ion are counted as coordinating[1]. (A-B) We performed MD simulations of TREK-1 and found that carbonyl oxygen atoms of I267 do not perfectly coordinate the K^+ ion during the entire simulation time, a fact that might explain why TREK-1^{I267T} is less selective for K^+. Distance measurement from amino acid 267 to the K^+ ion placed in S_2 site of the selectivity filter during 18 ns MD simulation from (A) wild-type TREK-1 and (B) I267T. The cut-off for a K^+ coordination site of 3.5 Å is indicated with a dotted line. (C-E) In addition, during the MD simulation in the presence of Na^+ the carbonyl group of the TREK-1 I267 residue of one subunit turns out from the SF (D) and water coordinates the Na^+ instead. This increased presence of water in the SF of TREK-1 (E) might explain why Na^+ can more easily pass the SF of TREK-1^{I267T}. (C) Distance measurement from amino acid 267 to the Na^+ ion placed in S_2 site of the selectivity filter during 18 ns MD simulation from wild-type TREK-1 shows a K^+ cage of oxygen atoms of the filter to bind the Na^+ ions, which probably does not allow the ion to pass trough the selectivity filter. Note that the smaller distances for Na^+ in a K^+ channel selectivity filter have been described previously.[2,3] (D) Distance measurement from amino acid 267 to the Na^+ ion placed in S_2 site of the selectivity filter during 18 ns MD simulation from I267T. At 4.2 ns the amino acid I267T from subunit B (lower panel) turns out of the selectivity filter and allows water to be located in the selectivity filter (not illustrated). (E) Amount of water molecules around Na^+ placed in S_2 site of the selectivity filter in wild-type TREK-1 (*black* lines) and TREK-1^{I267T} (*blue* lines) shows that I267T can be coordinated by more water molecules than wild-type TREK-1.

1. Wang, Y., Chamberlin, A.C. & Noskov, S.Y. Molecular strategies to achieve selective conductance in NaK channel variants. *J. Phys. Chem. B.* **118**, 2041-2049 (2014).
2. Shrivastava, I.H., Tieleman, D.P., Biggin, P.C. & Sansom, M.S. K^+ versus Na^+ ions in a K^+ channel selectivity filter: a simulation study. *Biophys. J.*, **83**, 633-645 (2002).
3. Tieleman, D.P., Biggin, P.C., Smith, G.R. Sansom M.S.P.. Simulation approaches to ion channel structure-function relationships. *Q. Rev. Biophys.* **34**, 473–561 (2001).

Appendix Fig S5

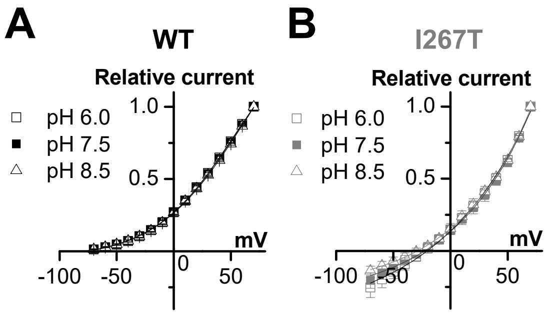

Appendix Fig S5. Increased depolarization under extracellular acidification of the TREK-1^{I267T} mutant.
(A) Average relative current amplitudes plotted against different potentials at pH_o 8.5, pH_o 7.5 and pH_o 6.0 for TREK-1 and (B) the TREK-1^{I267T} mutant. Data are presented as mean ± SEM.

Appendix Fig S6

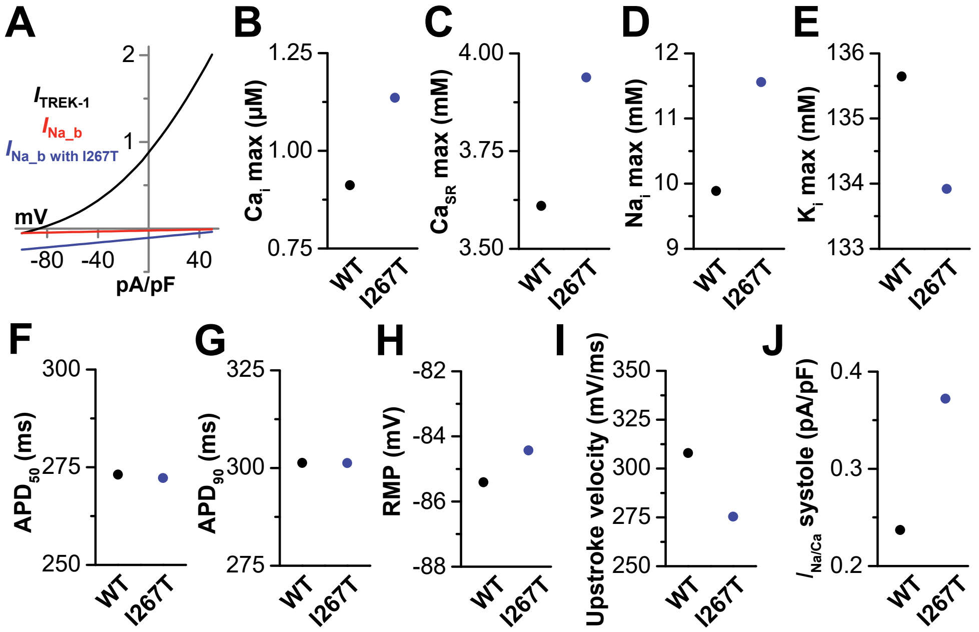

Appendix Fig S6. Effects of TREK-1^{I267T} in a computational action potential model of human ventricular cardiomyocytes.

The computational model was based on the human epicardial ventricular ionic model of ten Tusscher and Panfilov (ten Tusscher & Panfilov 2006, PMID: 16565318). (A) Current-voltage relationship of a leak potassium current based on the electrodiffusion theory (I_{TREK-1}, black line) with a current amplitude of 1.5 pA/pF at +30 mV, based on TREK-1 measurements by Bodnár *et al.* (Bodnár *et al*, 2015, PMID: 25539776) in rat ventricular cardiomyocytes. The red line shows the sodium background current as described in the ten Tusscher model (I_{Na_b}) and the blue line illustrates the increased sodium background current, based on the assumption that in the I267T mutant 20% of TREK-1 channels are conductive to sodium ions ($I_{Na\_b\ with\ I267T}$) (calibrated at -15 mV, see Appendix Material and Methods). The mutation causes in our model a 5.75-fold increase in the sodium background current. (B)-(J) illustrate the predictions of the model with (B) an increase in maximum intracellular calcium concentration of 25%, (C) maximum sarcoplasmic reticulum calcium concentration of 9%, (D) maximum intracellular sodium concentration of 17%, (E) a decrease in maximum intracellular potassium concentration of 1.3%, (F) a slight reduction of APD_{50} of 1 ms, (G) an almost unchanged APD_{90}, (H) a mild depolarization in the resting membrane potential of 1 mV, (I) a decreased action potential upstroke velocity of 10.5%, and (J) increased outward currents of the sodium calcium exchange current during the systole of 60.9%, reflecting an increased Ca^{2+} influx in the reverse mode.

Appendix Fig S7

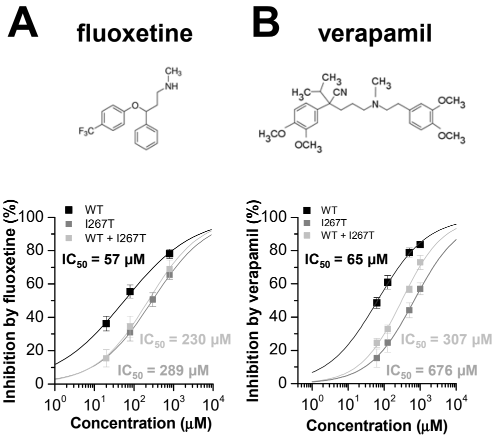

Appendix Fig S7. TREK-1 is blocked by verapamil and reduced blocker affinities for the I267T mutant.
(A) Chemical structure of fluoxetine and IC_{50} measurements of TREK-1 (n = 9, *black* line and IC_{50}), TREK-1^{I267T} (n = 8; *dark gray* line and IC_{50}) and TREK-1 co-expressed with TREK-1^{I267T} (n = 8, *light gray* line and IC_{50}). (B) Chemical structure of verapamil and IC_{50} measurements of TREK-1 (n = 5, *black line* and IC_{50}), TREK-1^{I267T} (n = 15, *dark gray* line and IC_{50}) and TREK-1 co-expressed with TREK-1^{I267T} (n = 5, *light gray* line and IC_{50}). Data are presented as mean ± SEM.

Appendix Fig S8

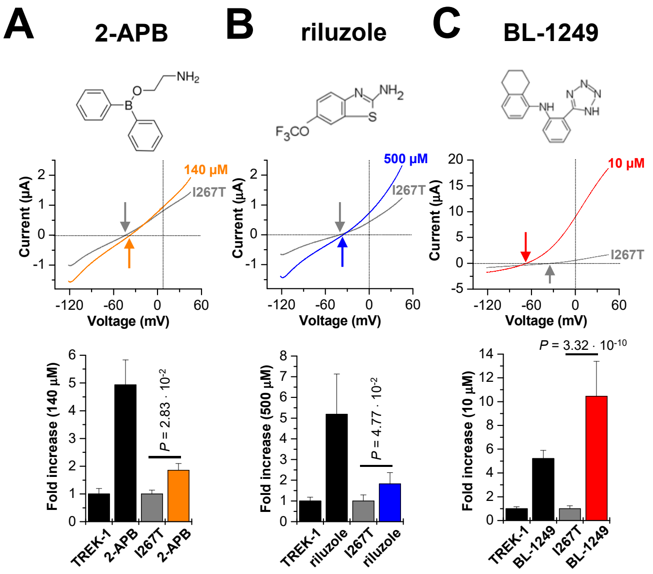

Appendix Fig S8. Reduced affinity of TREK-1^{I267T} to TREK activators and rescue of TREK-1^{I267T} by BL-1249.

(A) Chemical structure of 2-APB and representative current recordings of I267T before and after application of 140 µM 2-APB. The arrows indicate an additional mild depolarization upon TREK-1^{I267T} activation. The lower panel illustrates the fold-increased in TREK-1 (n = 5) and TREK-1^{I267T} (n = 4) currents by 140 µM 2-APB, analyzed at +40 mV. (B) Chemical structure of riluzole and representative current recordings of TREK-1^{I267T} before and after application of 500 µM riluzole. The arrows indicate an additional mild depolarization upon TREK-1^{I267T} activation. The lower panel illustrates the fold-increased in TREK-1 (n = 4) and TREK-1^{I267T} (n = 4) currents by 500 µM riluzole, analyzed at +40 mV. (C) Chemical structure of BL-1249 and representative current recordings of TREK-1^{I267T} before and after application of 10 µM BL-1249. The arrows indicate a pronounced hyperpolarization upon TREK-1^{I267T} activation upon activation by BL-1249. The lower panel illustrates the fold-increased in TREK-1 (n = 8) and TREK-1^{I267T} (n = 8) currents by 10 µM BL-1249, analyzed at 0 mV. Data are presented as mean ± SEM. *, p<0.05; ***, p<0.001, unpaired Student´s T-Test.

Appendix Fig S9

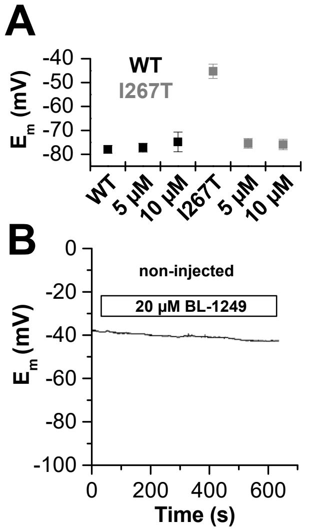

Appendix Fig S9. BL-1249 hyperpolarizes the membrane potential of TREK-1^{I267T} expressing oocytes.

(A) Analysis of the E_m of TREK-1 (*black*) or TREK-1^{I267T} (*gray*) expressing oocytes, before and after application of 5 and 10 µM BL-1249. Data are presented as mean ± SEM. (B) Representative control experiment illustrating the membrane potential (E_m) of a non-injected oocyte during application of high concentrations of BL-1249 (20 µM).

Appendix Fig S10

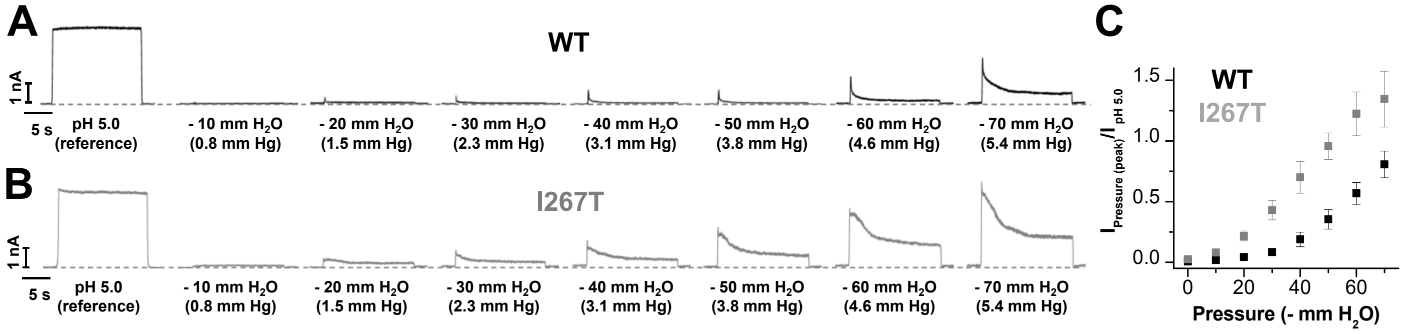

Appendix Fig S10. Increased stretch-sensitivity of TREK-1^{I267T} channels.
(A) Example traces from inside-out giant patches showing the pressure-dependent activation of wild-type TREK-1 currents for the indicated negative pressure values in comparison to a maximal intracellular pH activation (pH_i 5.0; reference) at a continuous potential of +40 mV. (B) Representative example for the TREK-1^{I267T} mutant, exhibiting an increased stretch-sensitivity over the same pressure range. (C) Summary of the stretch-activated instantaneous peak currents evoked by the indicated pressure values for wild-type TREK-1 (*black* squares, n = 5) and TREK-1^{I267T} (*gray* squares, n = 3). Data are presented as mean ± SEM.

Appendix Fig S11

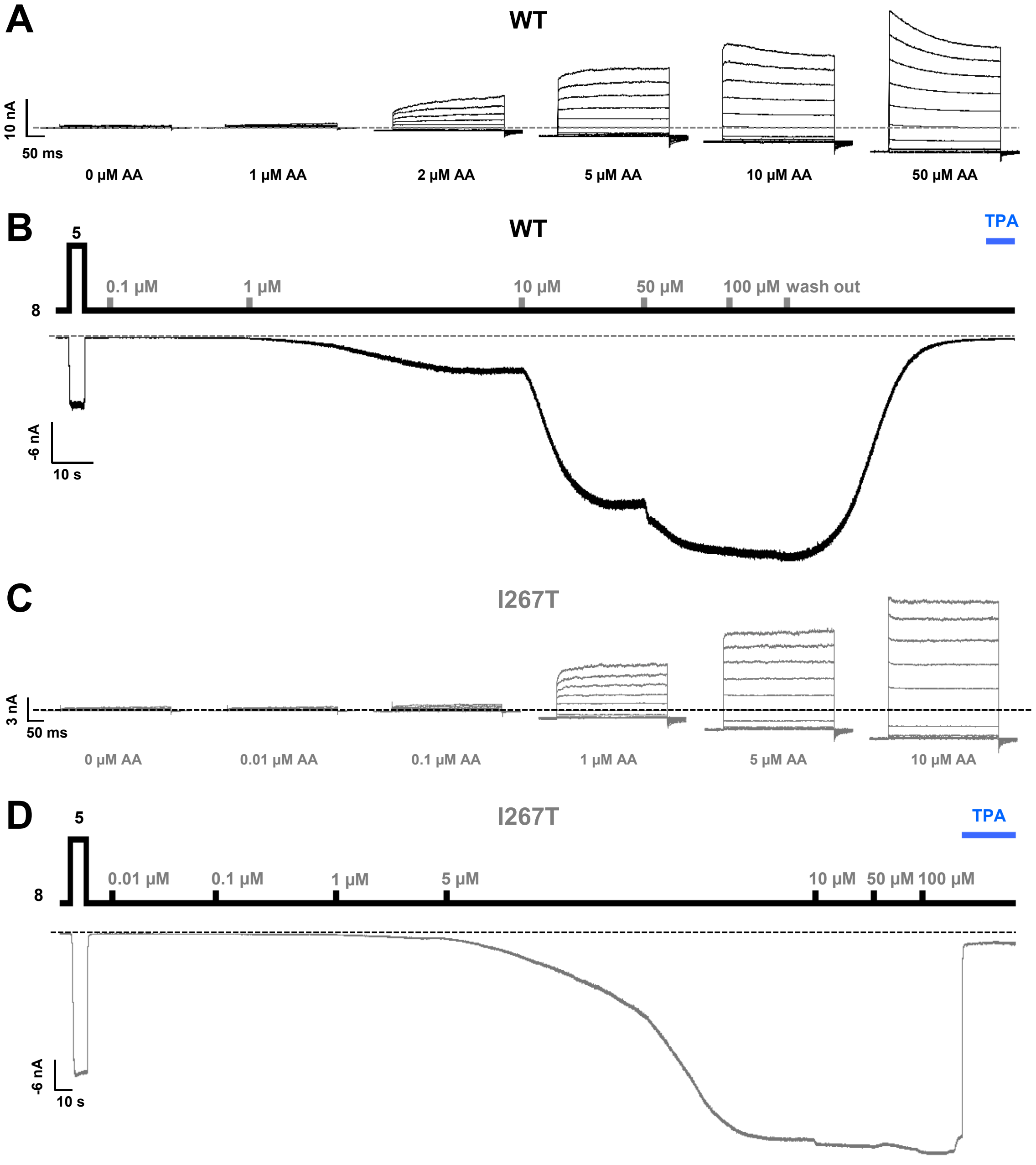

Appendix Fig S11. Enhanced arachidonic acid (AA) affinity of TREK-1^{I267T}.
(A) Representative wild-type TREK-1 current responses to a 300 ms voltage pulse family from -100 to +100 mV with 20 mV increments from a holding potential of -80 mV recorded from giant patches showing the dose-dependent activation by indicated concentrations of intracellular applied AA. (B) Example trace of the same activation at a continuous potential of -80 mV with the indicated AA concentrations. (C,D) Similar current responses to the voltage pulse family and at a continuous pulse of -80 mV for TREK-1^{I267T} and indicated AA concentrations. TPA (1 mM) indicates the specific block of K_{2P} channel currents by tetrapentylammonium.

Appendix Fig S12

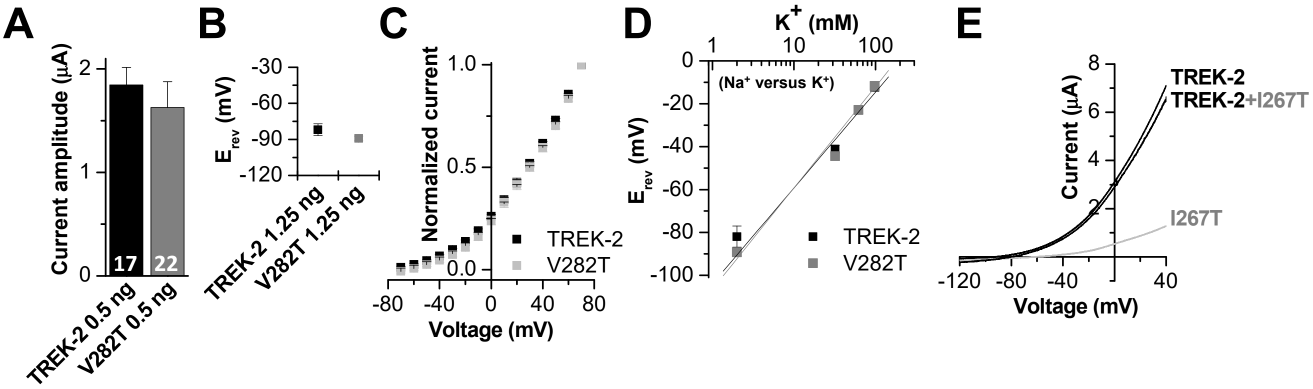

Appendix Fig S12. The I267T equivalent V282T mutation in TREK-2 does not induce a sodium leak and TREK-1^{I267T} does not induce a sodium leak in heteromers with TREK-2.

(A) Average current amplitudes recorded at +40 mV for oocytes injected with TREK-2 (0.5 ng/oocyte) or the I267T equivalent mutant V282T in TREK-2 (0.5 ng/oocyte). Currents were recorded 48 hours after cRNA injection, applying a ramp protocol for 3.5 s rising from -120 mV to +40 mV, from a holding potential of -80 mV. Numbers of experiments are indicated within the bar graph. (B) Mean reversal potential (E_{rev}) of oocytes injected with 1.25 ng of TREK-2 (*black*) or TREK-2^{V282T} (*gray*). (C) Normalized current-voltage relationship of wild-type TREK-2 and TREK-2^{V282T}. (D) Analysis of the E_{rev} of TREK-2 (*black*) and the V282T mutant (*gray*) as a function of the external potassium concentration, recorded by replacing extracellular NaCl to KCl. The slope of the extracellular potassium dependence is not significantly altered by V282T in TREK-2. (E) Representative current-voltage relationship measurements in oocytes injected with TREK-2, TREK-1^{I267T} or co-expressed TREK-2 with TREK-1^{I267T}. Data are presented as mean ± SEM. **, $p<0.01$; ***, $p<0.001$, unpaired Student´s T-Test.

Appendix Table S1

	Nucleotide	Protein	Annotation	Frequency in n=438 patients
intronic SNVs	c.46+16dup	-	-	2
	c.963+7A>C	-	rs80246222	2
synonymous SNVs	c.507C>T	p.Gly169=	rs17024342	3
	c.549C>T	p.Pro183=	rs41283140	1

Appendix Table S1. Polymorphic sites in the *KCNK2* gene.
In a patient cohort of 438 patients, a total of four polymorphic nucleotide sites were identified in *KCNK2*. All nucleotide variants were not considered as disease-causing because of benign pathogenicity prediction.

Appendix Table S2

Exome Data Analysis	
**Total sequence data (Gb)**	4.98
**Reads aligned (%)**	99.06
**Total number of reads aligned in pairs**	52,586,420
**Median coverage (0-250x)**	63.6 x
**0x coverage (%)**	0.27
**$\geq$20x coverage (%)**	88.14

Appendix Table S2. Overview of WES data in the proband with RVOT-VT.
Gb, Giga basepairs.

Appendix Table S3

	Gene	NCBI Accession No.	cDNA Level	Protein Level	Pathogenicity Prediction	MAF (%) in EVS
non-synonymous variants#	*KCNK2*	NM_001017425.2	c.800T>C	p.Ile267Thr	Damaging* (5/5)	0.02% (3/13003)
	RYR3	NM_001036.3	c.1753A>G	p.Ile585Val	Neutral** (4/5)	absent

Appendix Table S3. Single nucleotide variants with non-synonymous protein consequences identified after whole exome sequencing in a proband with RVOT-VT and results of different pathogenicity prediction tools (PolyPhen-2, SIFT, MutPred, SNPs&Go, SNAP).
*(5/5): fully concordant result of all pathogenicity prediction tools (PPT). * *(4/5): mainly concordant result for neutral effect of variant. MAF (%), minor allele frequency in percentage. EVS, Exomic Variant Server.

Appendix Table S4

Chr.	Gene	Chromosomal change g.	Transcript	Nucleotide change c.	Aminoacid change p.	Reference genome database	Annotation	MAF (%)
1	*CLCN6*	11884555A>G	NM_001286.3	593A>G	Glu198Gly	EVS	rs198400	0.085
1	*CLCNKB*	16371067G>T	NM_000085.4	80G>T	Arg27Leu	EVS	rs2015352	47.37
1	*GJB4*	35227010_35227013del	NM_153212.2	155_158del	Val52Alafs*55	EVS	rs146812843	3.43
1	*GJB3*	35250457C>T	NM_001005752.1	94C>T	Arg32Trp	EVS	rs1805063	2.31
1	*GJA4*	35260769C>T	NM_002060.2	955C>T	Pro319Ser	EVS	rs1764391	37.81
1	*CLCA2*	86900372C>G	NM_006536.5	916C>G	Gln306Glu	EVS	rs17409304	28.26
1	*CLCA1*	86952324A>G	NM_001285.3	1070A>G	Asn357Ser	EVS	rs2734705	13.09
1	*CLCA4*	87045902_87045907	NM_012128.3	2634_2639del	Thr886_Pro887del	EVS	rs4001061	31.77
1	*CACNA1S*	201016296G>A	NM_000069.2	4615C>T	Arg1539Cys	EVS	rs3850625	8.93
1	*CACNA1S*	201052310A>T	NM_000069.2	1373T>A	Leu458His	EVS	rs12742169	27.46
2	*KCNS3*	18113623A>G	NM_002252.3	1348A>G	Thr450Ala	EVS	rs4832524	33.42
2	*SCN1A*	166892788C>T	NM_001165963.1	3199G>A	Ala1067Thr	EVS	rs2298771	27.68
2	*SCN7A*	167279922C>A	NM_002976.3	2874G>T	Met958Ile	EVS	rs6738031	24.45
2	*SCN7A*	167334085G>T	NM_002976.3	122C>A	Thr41Asn	EVS	rs7565062	29.83
2	*KCNE4*	223917983T>G	NM_080671.2	435T>G	Asp145Glu	EVS	rs12621643	35.62
3	*CRELD1*	9976159A>G	NM_001031717.3	37A>G	Met13Val	EVS	rs279552	0.85
3	*SCN10A*	38739574T>C	NM_006514.2	5137A>G	Met1713Val	Ensembl	rs6599241	multiple observations
3	*SCN10A*	38766675A>G	NM_006514.2	3218T>C	Val1073Ala	EVS	rs6795970	29.75
3	*SCN10A*	38766760G>T	NM_006514.2	3133C>A	Pro1045Thr	EVS	rs73062575	2.27
3	*CACNA2D2*	50513613C>T	NM_001174051.1	224G>A	Arg75Gln	EVS	rs41291734	2.32
3	*KCNAB1*	155991410C>G	ENST00000475456	49C>G	Arg17Gly	dbSNP	rs13093003	10.9
3	*KCNMB3*	178968634C>T	NM_014407.3	157G>A	Ala53Thr	EVS	rs7645550	38.4
3	*KCNMB3*	178968660T>C	NM_014407.3	131A>G	Asp44Gly	EVS	rs1170672	9.41
6	*KCNK17*	39282036T>C	NM_031460.3	61A>G	Ser21Gly	EVS	rs10947804	34.66
7	*CLCN1*	143043240C>T	NM_000083.2	2180C>T	Pro727Leu	EVS	rs13438232	42.33
9	*CACNA1B*	140777306C>G	NM_000718.3	501C>T	Asn167Lys	Ensembl	rs4422842	multiple observations
11	*ABCC8*	17414570C>T	NM_000352.3	4714G>A	Val1572Ile	EVS	rs8192690	5.54
13	*GJA3*	20716533G>T	NM_021954.3	895C>A	Leu299Met	EVS	rs968566	3.16
14	*KCNK13*	90650514C>T	NM_022054.2	394C>T	Leu132Phe	EVS	rs55730840	0.62
15	*RYR3*	33873751G>A	NM_001036.3	1480G>A	Val494Ile	EVS	rs2077268	15.73
15	*RYR3*	33954652C>T	NM_001036.3	4921C>T	Arg1641Cys	EVS	rs4780144	13.19
15	*RYR3*	34137086_34137088del	NM_001036.3	13320_13322del	Glu4441del	EVS	rs149087920	13.51
16	*CACNA1H*	1252369C>T	NM_021098.2	1919C>T	Pro640Leu	EVS	rs61734410	22.35
16	*CACNA1H*	1254369C>T	NM_021098.2	2362C>T	Arg788Cys	EVS	rs3751664	7.38
16	*CACNA1H*	1270162G>A	NM_021098.2	6230G>A	Arg2077His	EVS	rs1054645	35.32
17	*KCNJ12*	21318867G>A	NM_021012.4	213G>A	Met71Ile	dbSNP	rs73979893	50
17	*KCNJ12*	21319079C>A	NM_021012.4	425C>A	Thr142Asn	Ensembl	rs76518282	multiple observations
17	*KCNJ12*	21319171G>A	NM_021012.4	517G>A	Asp173Asn	Ensembl	rs73313922	multiple observations
17	*KCNJ12*	21319208C>T	NM_021012.4	554C>T	Ala185Val	Ensembl	rs73979896	multiple observations
17	*KCNJ12*	21319230G>C	NM_021012.4	576G>C	Gln192His	Ensembl	rs1657742	multiple observations
17	*KCNJ12*	21319285C>T	NM_021012.4	631C>T	Leu211Phe	Ensembl	rs72846667	multiple observations
17	*KCNJ12*	21319369G>A	NM_021012.4	715G>A	Glu299Lys	Ensembl	rs77048459	multiple observations
17	*KCNJ12*	21319682C>T	NM_021012.4	1028C>T	Ser343Leu	Ensembl	rs80203231	multiple observations
17	*KCNJ12*	21319943A>G	NM_021012.4	1289A>G	Glu430Gly	Ensembl	rs5021699	multiple observations
17	*KCNH6*	61623052C>T	NM_030779.2	2774C>T	Thr925Met	EVS	rs35819807	21.87
17	*SCN4A*	62020348T>C	NM_000334.4	4126A>G	Asn1376Asp	EVS	rs2058194	40.07
19	*GDF1*	18980172G>A	NM_001492.4	353C>T	Ala/Val	dbSNP	rs4808863	28.4
19	*KCNA7*	49573438A>G	NM_031886.2	1253T>C	Met418Thr	EVS	rs1017219	38.53
20	*KCNK15*	43379264A>C	NM_022358.3	778A>C	Thr260Pro	EVS	rs6073538	46.29
20	*KCNK15*	43379268C>A	NM_022358.3	782C>A	Pro261His	dbSNP	rs13037900	10.1
20	*KCNK15*	43379454T>C	NM_022358.3	968T>C	Leu323Pro	EVS	rs13042905	40.00
20	*KCNS1*	43723627T>C	NM_002251.3	1465A>G	Ile489Val	EVS	rs734784	48.87
20	*KCNB1*	47989527C>T	NM_004975.2	2570G>A	Ser857Asn	EVS	rs34280195	0.75
20	*KCNB1*	47990250G>C	NM_004975.2	1847C>G	Thr616Ser	EVS	rs2229006	1.43
20	*KCNQ2*	62038277T>G	NM_172107.2	2339A>C	Asn780Thr	EVS	rs1801475	40.13
21	*KCNJ15*	39671476G>A	NM_002243.3	293G>A	Gly98Asp	EVS	rs2230033	40.29
22	*CACNA1I*	40058186A>G	NM_021096.3	3118A>G	Ile1040Val	EVS	rs136853	35.89

Appendix Table S4. Heterozygous single nucleotide variants (SNVs) within genes from an in-house gene panel (CARDIO Panel) obtained after WES and presence of SNV in control population databases (EVS, dbSNP, Ensembl Gene Browser).
Chr., Chromosome; MAF, minor allele frequency; EVS, Exome Variant Server.

Appendix Supplementary Methods

Computational single cell modeling

The human epicardial ventricular electrophysiology model of ten Tusscher and Panfilov (ten Tusscher & Panfilov, 2006, PMID: 16565318) was used to describe the effects of the TREK-1^{I267T} mutation on cardiac cellular behavior. As no ionic current formulation for I_{TREK} was present in this model, we decided to adjust the conductivity of the background sodium current accordingly. The long-term effects of this approach on ion concentrations are expected to be the same compared to a more accurate model of I_{TREK}. For the adjustment of the conductivity of the background sodium current, data from I_{TREK} in rat ventricular myocytes was considered. Bodnár *et al.* (Bodnár *et al*, 2015, PMID: 25539776) measured a TREK current of 1.5 pA/pF at +30 mV. We used this data to fit the permeability (P_{TREK}) of the electrodiffusion current equation developed by Goldman, Hodgkin and Katz:

$$I_{\mathrm{TREK}} = P_{\mathrm{TREK}} \frac{F^2 V_m}{RT} \frac{[K]_i - [K]_o exp\left(^{-FV_m}/_{RT}\right)}{1 - exp\left(^{-FV_m}/_{RT}\right)}$$

with F the Faraday constant, R the gas constant, T the absolute temperature, V_m the transmembrane voltage, and the concentration at the entrance and the exit of the pore $[K]_i$ and $[K]_o$, respectively. A ventricular I_{TREK} should have at -15 mV (a potential between the E_{Na} and E_K) an amplitude of about 0.63 pA/pF. A respective sodium inward leak due to TREK-1^{I267T} at -15 mV was assigned with 20% of the amplitude and the opposite polarity (-0.13 pA/pF). We chose 20% as our recordings revealed that the TREK-1^{I267T} has a sodium permeability of around 21%. The amplitude of -0.13 pA/pF was reached when the conductivity of the background current was set to g_{bNa}=0.0013705 nS/pF. In order to model TREK-1^{I267T}, this conductance was added to the standard value of the model and the total potassium background current conductivity was g_{bNa}=0.0016605 nS/pF.
The model was calculated for 1000 beats at a rate of 1 Hz to ensure steady state behavior for both the control model and the model including the increased background sodium current (TREK-1^{I267T}). An integration time step of 10 μs in combination with the Rush-Larsen method for the gating variables and the forward Euler method for all other variables was used.
